# Severe pulmonary hypertension associated with lung disease is characterised by a loss of small pulmonary vessels on quantitative computed tomography

**DOI:** 10.1183/23120541.00503-2021

**Published:** 2022-05-16

**Authors:** Dheyaa Alkhanfar, Yousef Shahin, Faisal Alandejani, Krit Dwivedi, Samer Alabed, Chris Johns, Allan Lawrie, A.A. Roger Thompson, Alexander M.K. Rothman, Juerg Tschirren, Johanna M. Uthoff, Eric Hoffman, Robin Condliffe, Jim M. Wild, David G. Kiely, Andrew J. Swift

**Affiliations:** 1 Dept of Infection, Immunity and Cardiovascular Disease, University of Sheffield, Sheffield, UK; 2 INSIGNEO, Institute for In Silico Medicine, University of Sheffield, Sheffield, UK; 3 Dept of Clinical Radiology, Sheffield Teaching Hospitals, Sheffield, UK; 4 Sheffield Pulmonary Vascular Disease Unit, Royal Hallamshire Hospital, Sheffield Teaching Hospitals NHS Foundation Trust, Sheffield, UK; 5 VIDA Diagnostics Inc., Coralville, IA, USA; 6 Dept of Computer Science, University of Sheffield, Sheffield, UK; 7 Dept of Radiology, University of Iowa, Iowa City, IA, USA; 8 These authors contributed equally

## Abstract

**Background:**

Pulmonary hypertension (PH) in patients with chronic lung disease (CLD) predicts reduced functional status, clinical worsening and increased mortality, with patients with severe PH-CLD (≥35 mmHg) having a significantly worse prognosis than mild to moderate PH-CLD (21–34 mmHg). The aim of this cross-sectional study was to assess the association between computed tomography (CT)-derived quantitative pulmonary vessel volume, PH severity and disease aetiology in CLD.

**Methods:**

Treatment-naïve patients with CLD who underwent CT pulmonary angiography, lung function testing and right heart catheterisation were identified from the ASPIRE registry between October 2012 and July 2018. Quantitative assessments of total pulmonary vessel and small pulmonary vessel volume were performed.

**Results:**

90 patients had PH-CLD including 44 associated with COPD/emphysema and 46 with interstitial lung disease (ILD). Patients with severe PH-CLD (n=40) had lower small pulmonary vessel volume compared to patients with mild to moderate PH-CLD (n=50). Patients with PH-ILD had significantly reduced small pulmonary blood vessel volume, compared to PH-COPD/emphysema. Higher mortality was identified in patients with lower small pulmonary vessel volume.

**Conclusion:**

Patients with severe PH-CLD, regardless of aetiology, have lower small pulmonary vessel volume compared to patients with mild–moderate PH-CLD, and this is associated with a higher mortality. Whether pulmonary vessel changes quantified by CT are a marker of remodelling of the distal pulmonary vasculature requires further study.

## Abstract 2

**In patients with pulmonary hypertension associated with lung disease, reduced small pulmonary vessel volume on quantitative CT imaging identifies patients with severe pulmonary hypertension** https://bit.ly/35bbEDu

## Introduction

Pulmonary hypertension (PH) in association with chronic lung disease (PH-CLD) and or hypoxia is associated with reduced functional status and increased mortality. It is most commonly seen in COPD/emphysema and interstitial lung disease (ILD). PH-CLD in this study included both the COPD/emphysema and ILD disease entities. For the majority of patients with PH-CLD, mean pulmonary arterial pressure (mPAP) elevation at right heart catheterisation (RHC) is usually mild to moderate (21–34 mmHg) and reflects the severity of underlying lung disease. However, a small proportion of patients have severe PH with mPAP ≥35 mmHg [1]. These patients are characterised by better preserved spirometry, normocapnia or hypocapnia and a significant reduction in gas transfer (diffusing capacity of the lung for carbon monoxide (*D*_LCO_)) [2, 3]. Given the poor prognosis of such patients there is increasing interest in conducting trials of pulmonary vasodilator therapy. However, the conduct of such trials is currently hampered by the heterogeneous nature of patients with PH-CLD where a number of mechanisms may contribute to pulmonary artery pressure elevation. An imaging biomarker that could aid improved phenotyping of the extent of vascular involvement in lung disease would be helpful.

CT imaging of the thorax has diagnostic utility and is recommended in the assessment of patients with suspected PH [4]. CT allows the qualitative visualisation and quantitative evaluation of the severity of lung parenchymal changes [4–6]. In addition, it can be used to assess the likelihood of PH. Typically, pulmonary arterial size [7, 8] is used to assess for the presence of PH. Moreover, where contrast is given, multiparameter models combining additional morphological characteristics including right ventricular tract hypertrophy and ventricular septal position improve diagnostic accuracy [9, 10], Automatic 3D extraction of pulmonary vessels from CT pulmonary angiograms has also been used to assess for the severity of PH [11]. However, whether the patterns of pulmonary vascular involvement in COPD/emphysema and ILD differ and how this relates to the severity of PH and lung parenchymal involvement is not known. Extraction of pulmonary arterial measurements from CT is an emerging approach [12, 13]. Quantitative evaluation of pulmonary vessel cross-sectional area has been shown previously to relate to PH severity in COPD [14], and thus there is potential for the use of CT vessel parameters to identify patients with more severe pulmonary vessel remodelling.

The primary aim of this study was to examine the differences in small pulmonary vessels in patients with and without severe PH in CLD. The secondary aim was to determine the differences in small pulmonary vessels in patients with COPD/emphysema or ILD, with and without PH.

## Methods

### Patients

Patients undergoing systematic assessment for suspected PH were identified from the ASPIRE registry between October 2012 and February 2018. Patients were required to have undergone CT pulmonary angiogram (CTPA), lung function testing (PFT) and RHC [15].

COPD may be defined as post-bronchiolar forced expiratory volume in 1 s (FEV_1_)/forced vital capacity (FVC) ratio ≤0.7, and according to the updated guidelines in 2010 of the National Institute for Health and Clinical Excellence (NICE), air flow obstruction is due to a combination of both airway and parenchymal damage [16], or due to significant emphysema. ILD was defined by the presence of CT features, reticular ground glass or/and honeycomb lung changes, in the absence of features of COPD/emphysema. High resolution computed tomography was used to assess the degree of emphysema/fibrosis, evaluated independently by two chest radiologists blinded to each other's findings and to clinical data [3]. COPD/emphysema was defined by either radiologically significant emphysema on CT scan or by spirometry in keeping with obstructive lung disease.

Patients were classified into four groups: 1) PH-COPD/emphysema, 2) PH-ILD, 3) COPD/emphysema or ILD without PH and 4) no PH and normal lung parenchyma on CT (control). Those with coexisting thromboembolic disease and combined pulmonary fibrosis and emphysema (CPFE) were excluded. Diagnoses were made following multidisciplinary team assessment as PH-COPD/emphysema or PH-ILD. Subsequently patients were subdivided based on the PH pressure threshold of 35 mmHg.

Approval for analysis of imaging data was granted by the local research ethics committee, and consent was waived for this retrospective database study (ref. c06/Q2308/8).

### Right heart catheterisation and PH severity

A balloon-tipped 7.5F thermodilution catheter (Franklin Lakes, Becton Dickinson, NJ, USA) was inserted *via* the internal jugular vein to obtain RHC measurements including mPAP, pulmonary capillary wedge pressure (PAWP) and cardiac output (CO). The thermodilution technique was used to measure CO. Pulmonary vascular resistance (PVR) was defined as (mPAP − PAWP)/CO. Measurements of pressure were averaged during quiet breathing. PH was defined as mPAP >20 mmHg and was further sub-classified into mild to moderate PH defined by mPAP 21–34 mmHg and severe PH defined by mPAP ≥35 mmHg [17].

### CT acquisition

CT scans were performed on a 64-slice multidetector computed tomography (MDCT) scanner (Light-Speed General Electric Medical Systems, Milwaukee, WI, USA), acquisition parameters: 120 kV, 100 mA with auto dose reduction, pitch 1, rotation time 0.5 s, field of view (FOV) 400×400 mm and slice thickness 0.625 mm; or a 320 detector-row CT system (Aquilion ONE/ViSION edition; Toshiba Medical Systems, Otawara, Japan), acquisition parameters: kV 120, modulated mA, pitch (standard pitch factor 0.813 and helical pitch 65), rotation time 0.275, FOV 500 L and slice thickness 0.5 mm. Intravenous contrast agents were administered with a dose of 100 mL (agent Ultravist 300; Bayer Schering, Berlin, Germany) at a rate of 5 mL·s^−1^. Contiguous slices were acquired during an inspiratory breath-hold.

### Quantitative CT pulmonary vessel analysis

Quantitative measurements from CTPA were extracted and computed automatically using Food and Drug Administration-approved lung quantitative imaging software (Apollo v2.0; VIDA Diagnostics, Coralville, IA, USA). This dedicated software was used to segment the lungs [18, 19] and the pulmonary vessels automatically with visual confirmation using an approach previously described [20, 21]. The total pulmonary vessel volume of each segment was measured as the volume of detectable arteries and veins, including vessel walls and luminal blood [22]. Total lung volume was the combined volumes of left and right lungs, measured in centimetres squared. Total vessel volume was the total vascular volume combined (arteries and veins), which is also measured in centimetres squared. The vascular mask files were resampled to an isotropic voxel size of 0.2 mm^3^ to allow for a comparison between scans taken at different resolutions. Small vessel volume (SVV) metrics represent the volume taken up by small vessels (arteries and veins combined) and were corrected according to body surface area (BSA), which was calculated using Mosteller's simplified calculation. We adjusted for BSA due to a known association between pulmonary arterial size and BSA [23]. Small pulmonary vessel volume metrics included three subcategories by maximal diameter thresholds: pulmonary vessels <0.8 mm, pulmonary vessels <1.2 mm and pulmonary vessels <1.6 mm.

### Qualitative lung scoring

Two radiologists scored the CT images, for the severity of lung parenchymal disease, independently, followed by a consensus read by the two radiologists and a final score recorded. A visual scoring system of the extent of lung diseases (emphysema/fibrosis)was used: <5%=minor, 5–25%=mild, 26–50%=moderate and >50%=severe [3, 24].

### Statistics

Statistical analysis was performed by using SPSS version 26.0 (SPSS, Chicago, IL, USA). A p-value <0.05 was considered significant. Histograms of CT parameters were used to check normality, and the data were normally distributed. Independent t-test was used to compare between the parameters in the groups. One-way ANOVA test with Bonferroni correction was used to determine whether there are statistically significant differences between the means of the parameters among the four groups. Pearson's correlation was used to detect associations between vessel parameters and both mPAP and PVR in each group.

Paired t-test was used to compare between the parameters in each group after dividing the cases according to mPAP into 21–34 mmHg and ≥35 mmHg. Receiver operating characteristic (ROC) curves were used to determine pulmonary vessel volume thresholds for the identification of patients with severe PH-CLD (mPAP ≥35 mmHg) in subgroups. The prognostic significance of these thresholds was assessed using Kaplan–Meier and multivariate Cox regression analysis.

## Results

### Patients

**FIGURE 1:**
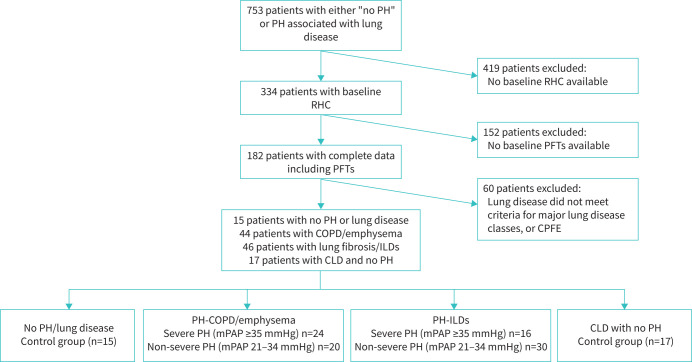
Study flowchart. PH: pulmonary hypertension; RHC: right heart catheterisation; PFTs: pulmonary function tests; mPAP: mean pulmonary artery pressure; ILD: interstitial lung disease; CPFE: combined pulmonary fibrosis emphysema syndrome; CLD: chronic lung disease.

122 patients met the study inclusion criteria including 44 patients with PH-COPD/emphysema, 46 patients with PH-ILD, 17 patients with no PH with chronic lung disease and 15 patients with no PH and no parenchymal lung disease (table 1). See figure 1 for a study flow diagram. Of the 90 patients with PH-CLD, 40 patients had severe PH. The demographics, results of lung function testing, pulmonary haemodynamics and CT vessel analysis are shown in table 1 and supplementary table S1. For patients with COPD (n=10) and ILD (n=7) with no PH (supplementary table S1), the mean±sd mPAP and BSA-corrected SVVs were 17.8±3 mmHg and 16.9±3 mmHg and 32±8 mL·m^−2^ and 23±6 mL·m^−2^, respectively.

**TABLE 1:** Group comparison of computed tomography-derived vessel parameters in patients with mild to moderate PH (mPAP 21–34 mmHg) *versus* patients with severe PH (mPAP ≥35 mmHg) in PH-COPD/emphysema and PH-ILD

	**COPD/emphysema**		**ILD**	
	**Mild–moderate PH**	**Severe PH**	**Mild–moderate PH**	**Severe PH**
**Subjects n**	20	24	30	16
**Demographics**				
Age years	65±13	67±11	64±12	69±16
Sex %	60% female	50% male	63% female	56% male
WHO functional class (I/II/III/IV) n	0/3/16/1	0/0/18/6	1/4/23/1	0/0/10/6
**Right heart catheter data**				
mRAP, mmHg	6±4	12±6*	6±3	11±11*
mPAP, mmHg	27±5	50±10**	26±4	48±9**
PAWP, mmHg	11±3	15±7*	11±3	11±5
Cardiac output, L·min^−1^	5.75±1.14	4.44±1.65*	5.16±1.26	3.95±0.93*
Cardiac index, L·min^−1^·m^−2^	3.30±0.74	2.36±0.70**	2.81±0.73	2.09±0.53*
PVR, mmHg	233±90	709±405**	257±143	777±242**
*S*_aO_2__, %	95±2	93±4	97±2	93±3**
*S*_vO_2__, %	74±6	62±9**	70±4	63±10*
**ISWT - distance, m**	231±153	105±74**	228±152	70±85**
**Pulmonary function tests**				
FEV_1_, % pred	66±24	62±20	67±19	54±14*
FVC, % pred	94±21	81±18*	68±20	55±17*
FEV_1_/FVC ratio, %	55±11	58±16	77±9	78±9
*T*_LCO_, % pred	49.2±23.1	21.6±11.6	37.6±20.8	17.8±11.8*
**All vessel parameters**				
Pulmonary vessels <0.8 mm, mL·m^−2^	10.6±3.9	8.1±2.7*	6.3±3.3	4.4±2.3*
Pulmonary vessels <1.2 mm, mL·m^−2^	22.6±7.7	17.2±5.4*	13.2±6.5	9.4±4.9*
Pulmonary vessels <1.6 mm, mL·m^−2^	34.5±11	27±8*	20.7±9.4	15±7.5*
Lung volume, mL	2884±643	2395±524*	1841±559	1526±510
Total vessel volume, mL	91±17	79±21*	55±17	49±24

Data expressed as mean±sd unless otherwise indicated. PH: pulmonary hypertension; ILD: interstitial lung disease; mPAP: mean pulmonary arterial pressure; WHO: World Health Organization; mRAP: mean right atrial pressure; PAWP: pulmonary artery wedge pressure; PVR: pulmonary vascular resistance; *S*_aO_2__: oxygen saturation; *S*_vO_2__: mixed venous oxygen saturation; ISWT: incremental shuttle walk test; FEV_1_: forced expiratory volume in 1 s; FVC: forced vital capacity; *T*_LCO_: transfer capacity of the lung for the uptake of carbon monoxide (CO). *: significant change between mild to moderate PH and severe PH (p<0.05); **: significant change between mild to moderate PH and severe PH (p<0.01).

### Group comparisons

#### Major lung disease subtypes

There were no significant differences in the age and sex of major lung disease subtypes. Patients with PH-ILD had a lower volume of small pulmonary vessels and lower lung volumes compared to all other groups (p=0.001 compared to control, p=0.0001 compared to COPD, p=0.01 compared to CLD no PH) (see supplementary table S1).

#### Severe versus non-severe pulmonary hypertension in association with chronic lung disease

Patients with severe PH due to either COPD/emphysema or ILD had higher PVR, lower *S*_vO_2__, lower incremental shuttle walk test distance and lower *D*_LCO_ compared to patients with mild to moderate PH. Whereas there was no significant difference between FEV_1_/FVC ratio between patients with mild to moderate PH and severe PH, those with severe PH due to both COPD/emphysema and ILD had a lower FVC (table 1).

**FIGURE 2:**
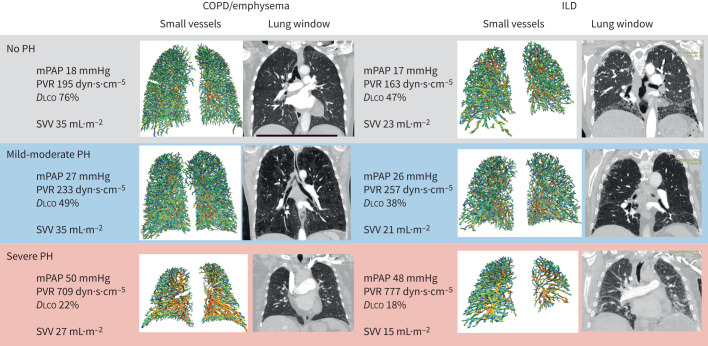
CT small vessels and coronal images from patients with COPD/emphysema and ILD with no PH, mild to moderate and severe PH. Representative images from patients with COPD/emphysema and ILD with mean values for mPAP and SVV for each group. ILD: interstitial lung disease; PH: pulmonary hypertension; mPAP: mean pulmonary artery pressure; PVR: pulmonary vascular resistance; *D*_LCO_: diffusing capacity of the lung for carbon monoxide; SVV: small vessel volume of vessels <1.6 mm.

Patients with PH-COPD with severe PH (n=24) (table 1) had a lower volume of small pulmonary vessels compared to mild to moderate PH for patients with both COPD/emphysema and ILD (table 1 and figure 2). Patients with ILD and severe PH (n=16) had lower pulmonary vessel volumes compared to patients with ILD and mild to moderate PH, and patients with no PH with or without lung disease (see table 2 and figure 2). At ROC analysis, optimal thresholds and ROC values shown in brackets for vessel volumes for the identification of severe PH-COPD for vessels with diameter <0.8 mm, 1.2 mm and 1.6 mm were 8.5 mL·m^−2^ (area under the curve (AUC)=0.69, p=0.02), 19 mL·m^−2^ (AUC=0.7, p=0.02) and 29 mL·m^−2^ (AUC=0.68, p=0.02) and for the identification of severe PH-ILD were 5 mL·m^−2^ (AUC=0.71, p=0.01) 11 mL·m^−2^ (AUC=0.69, p=0.04) and 16 mL·m^−2^ (AUC=0.71, p=0.01).

**TABLE 2:** Correlation of computed tomography-derived pulmonary parameters with mPAP and PVR in patients with PH and COPD/emphysema or ILD

	**PH–COPD/emphysema**		**PH–ILD**	
	**mPAP, R value/p-value**	**PVR, R value/p-value**	**mPAP, R value/p-value**	**PVR, R value/p-value**
**Subjects n**	44	42	46	44
**Pulmonary vessels <0.8 mm**	−0.37/0.01	−0.25/0.1	−0.37/0.01	−0.29/0.053
**Pulmonary vessels <1.2 mm**	−0.37/0.01	−0.26/0.1	−0.37/0.01	−0.30/0.051
**Pulmonary vessels <1.6 mm**	−0.35/0.02	−0.23/0.1	−0.37/0.01	−0.29/0.06
**Lung volume**	−0.32/0.03	−0.25/0.1	−0.32/0.03	−0.27/0.07
**Total vessel volume**	−0.17/0.2	−0.16/0.3	−0.16/0.2	−0.10/0.5

PH: pulmonary hypertension; ILD: interstitial lung disease; mPAP: mean pulmonary arterial pressure; PVR: pulmonary vascular resistance.

**FIGURE 3:**
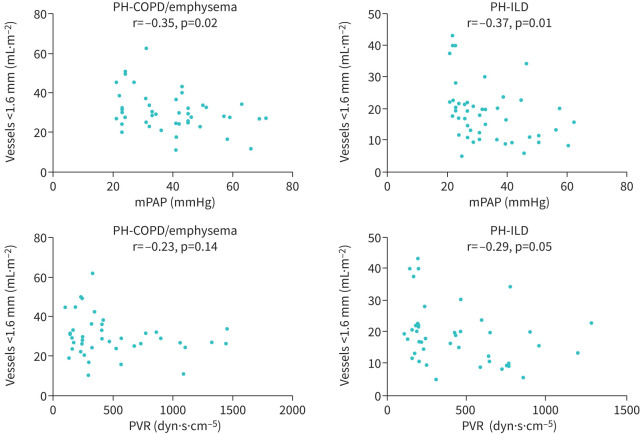
Correlation of pulmonary vessel volume <1.6 mm in diameter with mPAP and PVR in COPD/emphysema and ILD. PH: pulmonary hypertension; ILD: interstitial lung disease; mPAP: mean pulmonary artery pressure; PVR: pulmonary vascular resistance.

mPAP but not PVR was negatively correlated with the volume of small pulmonary vessels <0.8 mm, 1.2 mm and 1.6 mm in diameter in patients with COPD/emphysema and ILD, all p<0.05 (table 2 and figure 3). At regression analysis, the association of small pulmonary vessel volume with severe PH was found to be independent of age, sex and lung volume (<0.8 mm (p=0.001), <1.2 mm (p=0.031), <1.6 mm (p=0.004)).

### Associations with severity of lung disease

Patients with severe emphysema on CT had higher SVV metrics (p=0.01) and higher total vessel volumes (p=0.04) compared to patients with mild emphysema. In contrast, patients with moderate or severe ILD had lower SVV metrics (p=0.03) and lower total vessel volumes (p=0.04) compared to patients with mild ILD (table 3). In ILD all small pulmonary vessel volume metrics, total vessel volume and total lung volume correlated moderately with *D*_LCO_ (r=0.45–0.53), whereas no significant correlation was observed in COPD/emphysema. Moderate associations were identified between small vessel metrics and FVC in both COPD/emphysema and ILD (r=0.57–0.59). Lung volume on CT correlated strongly with FVC in ILD (r=0.74) and moderately in COPD/emphysema (r=0.54).

**TABLE 3:** Comparison of computed tomography derived vessel volumes according to the radiological severity of emphysema and interstitial lung disease (ILD)

	**Emphysema**			**ILD**		
**Variable**	**Mild**	**Moderate**	**Severe**	**Mild**	**Moderate**	**Severe**
**Pulmonary vessels <0.8 mm, mL·m^−2^**	7.6^+^	9.6	11.9^#^	7.9^¶^^,+^	4.9^#^	5^#^
**Pulmonary vessels <1.2 mm, mL·m^−2^**	16.2^+^	20	25.2^#^	16.6^¶^^,+^	10.3^#^	10.3^#^
**Pulmonary vessels <1.6 mm, mL·m^−2^**	25.3^+^	31.1	39.1^#^	25.7^¶^^,+^	16.5^#^	16.1^#^
**Total lung volume, mL·m^−2^**	2353	2639	3014	2107^¶^	1618^#^	1612
**Total vessel volume, mL·m^−2^**	71^¶^^,+^	90^#^	96^#^	70^¶^^,+^	48^#^	44^#^

^#^: significant difference compared to mild; ^¶^: significant difference compared to moderate; ^+^: significant difference compared to severe.

**TABLE 4:** Univariate and multivariate Cox proportional hazards regression analysis

	**Univariate**			**Adjustment for age and sex**			**Adjustment for age, sex and mPAP**		
	**B value**	**Hazard ratio**	**p-value**	**B value**	**Hazard ratio**	**p-value**	**B value**	**Hazard ratio**	**p-value**
**Age**	0.03	1.03	0.02	0.03	1.03	0.01	0.02	1.02	0.07
**Sex**	−0.45	0.63	0.09	−0.49	0.61	0.01	−0.16	0.85	0.58
**mPAP**	0.05	1.05	<0.01	0.05	1.05	<0.01	0.05	1.05	<0.01
**PVR**	0.003	1.003	<0.01	0.003	1.003	<0.01	0.003	1.003	<0.01
**Pulmonary vessels <0.8 mm**	−0.05	0.95	0.02	−0.09	0.92	0.03	−0.04	0.96	0.37
**Pulmonary vessels <1.2 mm**	−0.03	0.97	0.03	−0.04	0.96	0.03	−0.02	0.98	0.30
**Pulmonary vessels <1.6 mm**	−0.01	0.98	0.03	−0.03	0.97	0.05	−0.01	0.98	0.35

mPAP: mean pulmonary arterial pressure; PVR: pulmonary vascular resistance.

### Survival analysis

**FIGURE 4:**
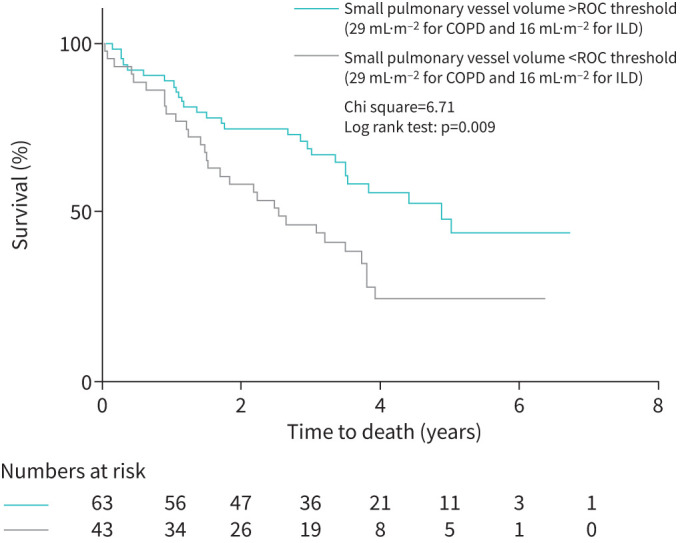
Kaplan–Meier plot showing patients with greater and lesser small vessel volumes (defined as small vessel diameter <1.6 mm) in patients with COPD/emphysema and ILD. ILD: interstitial lung disease; ROC: receiver operating characteristic curve.

In a combined group of patients with PH-COPD/emphysema and PH-ILD, reduced SVV was associated with worse survival than in patients with higher SVV: log rank chi-square 6.7, p=0.01 for vessels of diameter <1.6 mm (figure 4); log rank chi-square 4.9 and p=0.02 for vessels of diameter <1.2 mm; and log rank chi-square 2.4 and p=0.12 for vessels of diameter <0.8 mm. Adjusting for age and sex, the volume of small pulmonary vessels <0.8 mm (p=03), 1.2 mm (p=0.03) and 1.6 mm (p=0.05) were significant predictors of mortality. However, with adjustment for age, sex and mPAP, vessel volumes failed to remain a statistically significant prognostic factor (p=0.37, 0.30 and 0.35, respectively) (see table 4).

## Discussion

Using quantitative CT analysis on routinely performed CT pulmonary angiograms we have shown that the volume of small pulmonary vessels (<1.6 mm in diameter) is reduced in patients with severe PH compared to mild–moderate PH in both COPD/emphysema and ILD. A reduction in SVV was also associated with increased mortality.

In patients with PH-COPD/emphysema and PH-ILD, we have observed a negative association between mPAP at right heart catheterisation, and the volume of small pulmonary vessels <0.8, 1.2 and 1.6 mm in diameter, and this was independent of age, sex and lung volume. In addition, SVVs were significantly reduced in severe PH [25, 26] compared to mild to moderate PH in both COPD/emphysema and ILD. The significant negative association of SVV metrics with mPAP in both COPD/emphysema and ILD and the significant reduction in severe PH suggests that such a metric could potentially be used to identify patients with lung disease who may be more likely to have a vascular (pulmonary vascular phenotype) rather than ventilatory limit to exercise. A number of recent publications have demonstrated the importance of accurately phenotyping patients with PH-CLD and have highlighted the importance of haemodynamic assessment to identify patients with severe PH-CLD [24, 27–29]. Whether quantitative CT could be used to identify patients more likely to benefit from pulmonary arterial hypertension therapies and how this could be integrated with haemodynamic studies requires further study.

A prior study has assessed the relationship between the peripheral vessels by percentage small vessel cross-sectional area (%CSA, <5 mm^2^) and mPAP on CT in COPD and concluded that increased %CSA of small vessel areas was associated with mPAP elevation and was the optimal CT vessel parameter to detect severe PH in COPD [14]. In contrast, our study approach has evaluated the BSA indexed volume of small pulmonary blood vessels which is not adjusted for lung volume and has shown that small vessel volume is negatively associated with elevated mPAP. This apparent discrepancy may reflect that %CSA of small pulmonary arteries is scaled by lung volume. Our approach evaluated the absolute volume of small pulmonary blood vessels which is not adjusted for lung volume.

The cross-sectional areas of small vessels have previously been shown to be strongly correlated with the extent of emphysema [29]. A study in ILD has demonstrated an association between total pulmonary vessel volume and functional measures of severity in IPF [30], and has shown an association between increased total pulmonary vessel volume and mortality [31]. In patients with PH an increase in PVR increases the size of proximal vessels and therefore an association between an increase in total pulmonary vessel volume and mortality would not be unexpected. In our study we focused on small pulmonary vessels and their association with mortality. Although we cannot assess for involvement of small pulmonary arterioles (which contribute most to an increase in resistance), we have hypothesised that measuring small pulmonary vessels may be a better reflection of the impact of the underlying lung disease on the pulmonary vasculature and a better reflection of more distal vascular lung involvement. The lowest values of small vessel and total vessel volumes were present in the PH-ILD. In PH-ILD we found a moderate positive association between SVV and lower *D*_LCO_, suggesting a potential link between loss of small vessels on CT and vascular involvement. This association was not found with PH-COPD/emphysema suggesting that the relationship between vascular involvement and PH in COPD/emphysema may be more heterogeneous with an elevated mPAP not necessarily a consequence of vascular involvement. When we compared the vessel volumes between the three severity scales of emphysema (mild, moderate and severe), we found that the severe emphysema associates with higher small pulmonary vessel volume compared to mild to moderate emphysema. In contrast, in patients with ILD, we found the converse, with patients with more severe parenchymal disease having a lower volume of blood in the small pulmonary vessels. These findings suggest that the impacts of COPD/emphysema and ILD on the pulmonary vasculature are very different, and we postulate that in ILD vascular involvement may be more uniform whereas in COPD/emphysema it is more heterogeneous. This shows that the relationship between severity of lung parenchymal changes with small pulmonary vessels differs between ILD and COPD/emphysema. However, our study has shown that consistently lower small pulmonary blood vessel volumes are found in patients with severe PH. Advances in the application of artificial intelligence to medical imaging may provide additional insights [32].

### Limitations/future directions

This is a retrospective study from a single centre. No separation of arteries and veins was made, and further work to evaluate the accuracy of AV separation in larger clinical cohorts would be desirable [33]. No quantitative lung density or texture analysis was performed. Such methods are not yet established for contrast enhanced CT, and this is an area for further research. The volume of small pulmonary vessels <0.8 mm failed to predict mortality; this size of vessel is at the limit of resolution of CT, and hence accurate quantification may be challenging.

### Conclusion

This study is the first to demonstrate that small pulmonary vessel volume is reduced in severe PH-CLD compared to mild to moderate PH-CLD. Whether this reflects more severe small vessel involvement and whether it could be used to identify patients more likely to benefit from interventions directed at the pulmonary vasculature require further study.

## Supplementary material

10.1183/23120541.00503-2021.Supp1 **Please note:** supplementary material is not edited by the Editorial Office, and is uploaded as it has been supplied by the author.
Supplementary material 00503-2021.SUPPLEMENT
