# Supplementary material for: Severe pulmonary hypertension associated with lung disease is characterised by a loss of small pulmonary vessels on quantitative computed tomography
Source: ERJ Open Res. 2022 May 16;8(2):00503-2021. doi: 10.1183/23120541.00503-2021 (PMC9108962; doi:10.1183/23120541.00503-2021)
Supplement: Supplementary file 1 [file 00503-2021.SUPPLEMENT.pdf]

Supplementary table 1 Demographic, right heart catheter, lung function and CT vessel data

**Variables**<br>**Units**	**No PH**<br>**No lung disease**<br>**(n=15)**	**No PH**<br>**Lung disease**<br>**(n=17)**<br>**(COPD n=10, ILD n=7)**	**All PH**<br><br>**(n=90)**	**PH**<br>**COPD and or emphysema**<br>**(n=44)**	**PH**<br>**ILD**<br><br>**(n=46)**
**Demographics**					
Age (years)	63 (11)	65 (10)	66 (13)	66 (12)	66 (14)
Sex (M/F) %	76% F	87% F	44% F	55% F	55% F
WHO functional class (I/II/III/IV) £	I (2) II (8) III (5) IV (0)	I (0) II (6) III (10) IV (1)	I (1) II (7) III (78) IV (14)	I (0) II (3) III (34) IV (7)	I (1) II (4) III (34) IV (7) £
**Right heart catheter data**					
mRAP (mmHg)	6 (3)	4 (3) #	8 (5)	9 (6) ^	7 (5)
mPAP (mmHg)	17 (2) #$	17 (3) #$	36 (13)	39 (14) *^	34 (12)*^
PAWP (mmHg)	9 (3) #	8 (4) #	12 (5)	13 (6) *^	11 (4)
Cardiac_output (L/min)	5.24 (1.80)	4.74 (1.67)	4.88 (1.43)	5.03 (1.57)	4.73 (1.28)
Cardiac index (CI) (L/min/m^2)	3.07 (0.93)	2.70 (0.79)	2.67 (0.80)	2.79 (0.85)	2.56 (0.74)
PVR (mmHg)	130 (39) #$	181 (104) #$	457 (344)	482 (382) *^	434 (307) *^
SaO_2 (%)	96 (2)	96 (2)	95 (3)	94 (3)	95 (3)
SVO_2 (%)	73 (5)	69 (4)	68 (9)	67 (10)	68 (7)
**ISWT - distance (m)**	296 (162) #	280 (177) #	169 (141)	158 (129) *^	181 (151)
**Pulmonary Function Tests (PFTs)**					
Percent predicted FEV^1 (%)	90 (13) #$	80 (14) #$	63 (20)	64 (22) *^	62 (18) *^
Percent predicted FVC (%)	94 (13) $	86 (20) $	75 (23)	87 (22) $	63 (20) *#^
FEV^1/FVC ratio	75 (3) #	74 (11) #	67 (15)	57 (14) *$^	77 (9) #
Percent predicted TLCO (%)	71 (17.5) #$	63.3 (27.6) #$	32.7 (21.5)	34.7 (22.6) *	30.6 (20.2) *^
**CT vessel parameters**					
Pulmonary vessels <0.8mm (ml/m^2)	9.4 (3.4) $	8.7 (2.8)$	7.4 (3.8)	9.2 (3.5) $	5.6 (3.1) *#^
Pulmonary vessels <1.2mm (ml/m^2)	19.8 (7.2) $	18.4 (5.8) $	15.7 (7.6)	19.6 (7) $	11.8 (6.1) *#^
Pulmonary vessels <1.6mm (ml/m^2)	30.3 (10.7) $	28.3 (8.1) $	24.4 (11.2)	30.4 (10.1) $	18.6 (9.1) *#^
Lung volume (ml/m^2)	2371 (604) $	2243 (495) $	2165 (738)	2618 (625) $	1720 (557) *#^
Total vessel volume (ml/m^2)	75 (24) $	69 (15) $	68 (25)	84 (19) $	53 (20) *#^

*Significant difference compared to no PH and no lung disease
#Significant difference compared to PH-COPD/emphysema.
$Significant difference compared to PH-ILDs.
^Significant difference compared to lung disease and no PH
£ 4 cases missing WHO functional class measurements

*WHO=world health organisation, mRAP=mean right atrial pressure, mPAP=mean pulmonary arterial pressure, PAWP=pulmonary artery wedge pressure, PVR=pulmonary vascular resistance, SaO_2=Oxygen saturation, SVO_2=Mixed

venous oxygen saturation, FEV^1=Forced Expiratory Volume in 1 second, FVC=Forced Vital Capacity, TLCO=transfer capacity of the lung for the uptake of carbon monoxide (CO).
